# Coronavirus surveillance in passerines reveals novel deltacoronaviruses in Eurasian tree sparrows with implications for One Health and livestock biosecurity

**DOI:** 10.1016/j.onehlt.2026.101560

**Published:** 2026-08-31

**Authors:** Urška Kuhar, Neža Mrzdovnik, Danijela Černe, Petra Šenica Kavčič, Tomi Trilar, Milka Vrecl Fazarinc, Modest Vengust

**Affiliations:** a University of Ljubljana, Veterinary Faculty, Gerbičeva 60, 1000 Ljubljana, Slovenia; b Slovenian Museum of Natural History, Prešernova 20, p.p. 290, SI – 1001 Ljubljana, Slovenia

**Keywords:** *Passer montanus*, Deltacoronavirus, Viral recombination, Synanthropic birds, Wildlife–livestock interface, Zoonotic potential, Cross-species transmission, Whole-genome sequencing

## Abstract

Coronaviruses (CoVs) are widespread RNA viruses infecting a broad range of avian and mammalian hosts. Although gammacoronaviruses and deltacoronaviruses (DCoVs) are common in wild birds, their presence in Eurasian passerines remains poorly understood. We screened 243 birds (35 species) at migratory stopover sites in Slovenia (2020−2021) using pan-coronavirus RT-PCR. Coronavirus RNA was detected only in four Eurasian Tree Sparrows (*Passer montanus*). Whole-genome sequencing yielded genomes of 26,017–26,018 bp with high internal conservation (99.95–99.98% identity).

Phylogenetic analysis revealed notable evolutionary incongruence: isolates were highly related to porcine DCoVs in the ORF1ab region (95.6–96.1% amino acid identity) but clustered with divergent avian DCoVs in the spike gene (75.7–76.8% identity). RDP5 analysis provided strong evidence for a large-scale recombination event (*p* = 1.17 × 10–43), consistent with a mosaic genomic architecture combining an ORF1ab region closely related to porcine DCoVs with an avian-associated spike gene. This genomic pattern highlights evolutionary connectivity among DCoVs associated with different host groups and the potential role of recombination in changes in host association.

The synanthropic behaviour and mobility of *P. montanus* facilitate contact with diverse hosts, making this species relevant for investigating DCoV ecology at wildlife–livestock interfaces. These findings represent the first genomic characterisation of DCoVs in *P. montanus* in Europe and support the inclusion of passerines in broader coronavirus surveillance. Genomic surveillance of underrepresented wild-bird hosts can improve our understanding of DCoV diversity, recombination, and evolution across wildlife–livestock interfaces.

## 1 Introduction

Coronaviruses (CoVs) are enveloped, single-stranded, positive-sense RNA viruses that infect a wide range of mammals and birds [1]. They are primarily associated with respiratory and gastrointestinal diseases and are transmitted via direct contact, aerosolized droplets, or contaminated surfaces [2]. The ability of CoVs to emerge from animal reservoirs and cross species barriers poses a significant threat to both human and animal health, as demonstrated by major outbreaks such as severe acute respiratory syndrome (SARS), Middle East respiratory syndrome (MERS), and COVID-19 in humans [3], [4], as well as porcine epidemic diarrhea virus (PEDV), which has caused widespread morbidity and mortality in piglets and considerable economic losses in the swine industry [5], [6]. While global attention has largely focused on the zoonotic risk of betacoronaviruses, deltacoronaviruses (DCoVs) are predominantly associated with avian hosts and represent an important component of coronavirus diversity in wild birds [7]. Their evolutionary significance is further highlighted by the demonstrated capacity of some DCoVs to cross the avian–mammalian species barrier [8]. Their high genetic plasticity, resulting from mutation and recombination, drives the diversification of DCoVs and, in some cases, may facilitate changes in host range [7], [9].

The family *Coronaviridae* is divided into four genera: *Alphacoronavirus*, *Betacoronavirus*, *Gammacoronavirus*, and *Deltacoronavirus*. While alphacoronaviruses and betacoronaviruses primarily infect mammals and are believed to have originated in bats, gammacoronaviruses and DCoV predominantly infect avian species, which serve as their natural reservoirs and primary gene sources [10]. Frequent detections of CoVs from multiple genera in wild bird populations highlight the role of avian hosts in coronavirus diversity and evolution and the importance of understanding viral circulation across ecological interfaces [11], [12].

DCoVs, circulate widely in avian hosts, while some lineages have also been associated with domestic animal infections, making their diversity and evolutionary dynamics relevant to wildlife surveillance at the animal interface. Transmission between domestic poultry and wild birds is well documented [13], [14], [15], indicating established pathways for viral exchange across ecological boundaries. While DCoVs are primarily avian pathogens, Porcine DCoV (PDCoV; HKU15) exemplifies avian-to-mammalian spillover; it likely originated from avian sources and can infect human and animal cells in vitro via the aminopeptidase N (APN) receptor [7], [10], [16]. The evolutionary success of DCoVs in crossing these barriers is largely due to their modular genome structure. In particular, the spike (S) gene can be exchanged through recombination, enabling rapid adaptation to novel host receptors [17]. Identifying such genomic changes in naturally circulating DCoVs is therefore important for understanding their evolution and host associations.

The world's largest bird migration system, the Palaearctic–African flyway, involves an estimated 2 billion passerines and near-passerines migrating annually between Europe and sub-Saharan Africa [18]. These wild birds serve as long-distance dispersal vectors for various pathogens. Passerines (Order *Passeriformes*) are of particular interest due to their high mobility, gregarious behaviour, and frequent use of human-modified environments [19]. However, their role as viral spreaders is not limited to natural migration; passerines are often subjected to illegal trapping and trade, especially in the Mediterranean region [20], [21]. This illicit activity facilitates intercontinental mixing of avian populations outside any formal biosecurity control, creating high-risk interfaces for viral exchange. Slovenia, situated at the crossroads of these migratory routes and illegal trade corridors, represents a strategic location for monitoring such dynamics. Despite their global movement ecology and importance to transboundary animal disease dynamics, passerines remain significantly underrepresented in coronavirus surveillance compared to waterfowl and poultry.

The aim of this study was to implement targeted surveillance for coronaviruses in Eurasian passerines and to contribute to understanding DCoV diversity in wild birds. Specifically, we sought to characterise the genomic architecture of DCoVs detected in *P. montanus* and investigate evidence of recombination. By analysing the evolutionary relationship between these viruses and known porcine and avian DCoVs, assessed their genomic and evolutionary relationships at the wildlife–livestock interface.

## 2 Materials and methods

### 2.1 Sample collection

Fresh fecal samples from passerines were collected opportunistically during routine bird-ringing activities in Slovenia during the autumn migrations of 2020 and 2021. Sampling was conducted at two traditional stopover sites (45°58′07.0″ N, 14°18′29.2″ E and 45°55′37.2″ N, 14°38′36.1″ E). Birds were captured using mist nets under the authorization of the Slovenian Environment Agency (permit no. 35601–8/2015–7) as detailed in the Ethics statement.

To minimize contamination and ensure biosecurity, each bird was placed in an individual sterile net bag. If defecation occurred during routine handling, fecal material was immediately transferred into sterile, RNase-free microcentrifuge tubes. Only birds from which fecal material was obtained were included in the study, resulting in a total of 243 individual avian fecal samples. Birds were not retained for additional time to obtain fecal samples. All birds were identified to species level and ringed as part of the ongoing bird-monitoring programme, in which individually numbered metal rings allow identification upon subsequent recapture or recovery and provide information on bird movements and migration. Field personnel (*n* = 6) adhered to strict biosafety protocols, including the use of FFP2 masks and the changing of sterile gloves between handling individual birds. Additionally, nasopharyngeal swabs were collected on each sampling day from all members of the field team who participated in bird handling on that day, to monitor for potential anthropogenic contamination of the avian samples. For each individual field team member (*n* = 6), swabs collected across sampling days were pooled before analysis, resulting in six pooled human samples. All samples were immediately flash-frozen in liquid nitrogen and subsequently stored at −80 °C until further analysis.

### 2.2 Pan-Coronavirus RT-PCR

A total of 249 samples (243 avian fecal and six human nasopharyngeal swabs) from apparently healthy individuals were screened for coronaviruses. Fecal samples were suspended in 1 mL RPMI-1640 medium (Gibco, Invitrogen) and incubated overnight at 4 °C. After centrifugation (1200 ×*g*, 10 min), supernatants were pooled in groups of four to optimise screening efficiency. Total RNA was extracted from 200 μL of the pooled suspension using the MagMAX™ CORE Nucleic Acid Purification Kit on the KingFisher Flex System (ThermoFisher, USA) according to the manufacturer's protocol.

Coronavirus RNA was detected using two pan-coronavirus RT-PCR assays with degenerate consensus primers targeting the RNA-dependent RNA polymerase (RdRp) gene, as previously described [22], [23]. Samples from positive pools were re-tested individually to identify the positive birds. PCR products were visualised using the QIAxcel capillary electrophoresis system (Qiagen, Germany), and the resulting amplicons were subjected to Sanger sequencing (Macrogen Europe, The Netherlands) for initial taxonomic confirmation. The prevalence of DCoV in *Passer montanus* was calculated as the proportion of positive individuals, with 95% confidence intervals (CI) estimated using the Clopper–Pearson exact method.

### 2.3 Whole-genome sequencing and bioinformatics analysis

Whole-genome sequencing was performed on the four RT-PCR–positive samples using the Illumina NovaSeq 6000 platform (Novogene Europe, UK). Total RNA extracted from the positive samples was used for library preparation via the RNA immunoprecipitation sequencing (RIP-seq) method, employing 2 × 150 bp paired-end chemistry.

Raw FASTQ reads were processed to merge overlapping paired-end reads and remove low-quality bases using BBMerge v38.37 [24]. The resulting high-quality merged reads were assembled de novo using SPAdes v3.13.1 [25]. To identify viral sequences, the assembled contigs were screened using DIAMOND BlastX [26] against the NCBI non-redundant protein database and taxonomically assigned using MEGAN6 [27].

Downstream analysis was conducted in Geneious Prime v2022.1.1. To ensure assembly accuracy, all sequenced reads for each sample were mapped back to the assembled contigs using the Geneious reference mapper to generate final consensus sequences. Open reading frames (ORFs) were predicted using the Geneious ORF Finder and annotated via multiple alignment in MAFFT v7.450 [28] relative to the *P. montanus* DCoV reference genome (GenBank: MG812375). Finalised sequences were deposited in GenBank under accession numbers PX204799–PX204802.

### 2.4 Phylogenetic and sequence diversity analysis

Reference genome sequences of representative porcine and avian DCoVs were retrieved from GenBank. Whole-genome nucleotide alignments were generated using MAFFT v7.450 [28], and pairwise nucleotide identities were calculated. Maximum-likelihood (ML) phylogenetic trees were reconstructed with PhyML v3 [29] and visualised in MEGA7 [30]. The best-fit nucleotide substitution model (GTR + R) was selected by PhyML-SMS using the Bayesian information criterion (BIC) [31]. Branch support was assessed with Shimodaira–Hasegawa approximate likelihood-ratio tests (SH-aLRT), with values ≥80 considered statistically robust

For the ORF1ab and S genes, amino acid sequences were aligned in MAFFT, and pairwise identities were determined. ML trees were constructed in PhyML v3 using the LG + R + F (ORF1ab) and WAG+R + F (S) models.

### 2.5 Recombination and structural modelling

Potential recombination signals were explored using RDP5 v5.23 [32] with default settings and Bonferroni-corrected *p* < 0.05 thresholds. Similarity plots and BootScan analyses were generated in SimPlot v3.5.1 [33] to validate crossover points (e.g., position 19,524) and parental lineages. Putative receptor-binding domains (RBDs) of the S protein were identified in silico using structural alignment with reference spike protein structures [34].

## 3 Results

### 3.1 Detection of CoV by RT-PCR

During the study period, fecal samples were collected from 243 individual birds representing 35 distinct species (Table 1). The most frequently sampled taxa were *Passer montanus* (*n* = 25), *Regulus regulus* (*n* = 22), *Prunella modularis* (*n* = 20), and *Hirundo rustica* (*n* = 19), along with various members of the *Acrocephalus* and *Sylvia* genera. Initial screening by RT-PCR targeting the RdRp gene detected coronavirus RNA in four pooled samples. All positive pools originated exclusively from Eurasian Tree Sparrows (*Passer montanus*). Subsequent individual testing of the samples within these pools confirmed four positive individuals. Initial sequencing of the PCR amplicons showed that these sequences belonged to the genus DCoV, and these four specimens were selected for in-depth whole-genome sequencing.

**Table 1:** Summary of avian species sampled and Deltacoronavirus (DCoV) detection.

Family	Species	Common Name	n	DCoV Positive
Passeridae	*Passer montanus*	Eurasian Tree Sparrow	25	4
	*Passer domesticus*	House Sparrow	2	0
Acrocephalidae	*Acrocephalus arundinaceus*	Great Reed Warbler	4	0
	*Acrocephalus schoenobaenus*	Sedge Warbler	11	0
	*Acrocephalus scirpaceus*	Reed Warbler	4	0
	*Acrocephalus palustris*	Marsh Warbler	1	0
Sylviidae	*Sylvia atricapilla*	Blackcap	10	0
	*Sylvia borin*	Garden Warbler	12	0
	*Sylvia communis*	Common Whitethroat	7	0
	*Sylvia curruca*	Lesser Whitethroat	2	0
Fringillidae	*Carduelis carduelis*	European Goldfinch	6	0
	*Carduelis chloris*	European Greenfinch	2	0
	*Serinus serinus*	European Serin	1	0
Muscicapidae	*Erithacus rubecula*	European Robin	19	0
	*Luscinia megarhynchos*	Common Nightingale	1	0
Hirundinidae	*Hirundo rustica*	Barn Swallow	19	0
Paridae	*Parus major*	Great Tit	9	0
	*Cyanistes caeruleus*	Blue Tit	1	0
	*Periparus ater*	Coal Tit	7	0
	*Lophophanes cristatus*	Crested Tit	1	0
	*Poecile palustris*	Marsh Tit	1	0
Phylloscopidae	*Phylloscopus collybita*	Common Chiffchaff	17	0
	*Phylloscopus trochilus*	Willow Warbler	2	0
Regulidae	*Regulus regulus*	Goldcrest	22	0
Turdidae	*Turdus merula*	Common Blackbird	18	0
	*Turdus philomelos*	Song Thrush	2	0
Prunellidae	*Prunella modularis*	Dunnock	20	0
Other Families	*Alcedo atthis*	Common Kingfisher	4	0
	*Dendrocopos major*	Great Spotted Woodpecker	1	0
	*Jynx torquilla*	Eurasian Wryneck	5	0
	*Locustella luscinioides*	Savi's Warbler	1	0
	*Locustella naevia*	Common Grasshopper Warbler	1	0
	*Motacilla cinerea*	Grey Wagtail	2	0
	*Oriolus oriolus*	Eurasian Golden Oriole	1	0
	*Troglodytes troglodytes*	Eurasian Wren	2	0
Total			243	4

No coronavirus RNA was detected in any of the six pooled nasopharyngeal samples from field personnel.

### 3.2 Genomic characterisation

Of the 25 *P. montanus* individuals tested, four were positive for DCoV, representing a localised prevalence of 16.0% (95% CI: 4.54%–36.08%). In contrast, no viral RNA was detected among the remaining 218 individuals from 34 other passerine species. We determined the complete genomes of the four Eurasian Tree Sparrow DCoV strains using Illumina sequencing, yielding lengths of 26,017 bp (strains DCoV73/2020, DCoV78/2020, and DCoV84/2020) and 26,018 bp (DCoV85/2020).

**Fig. 1:**
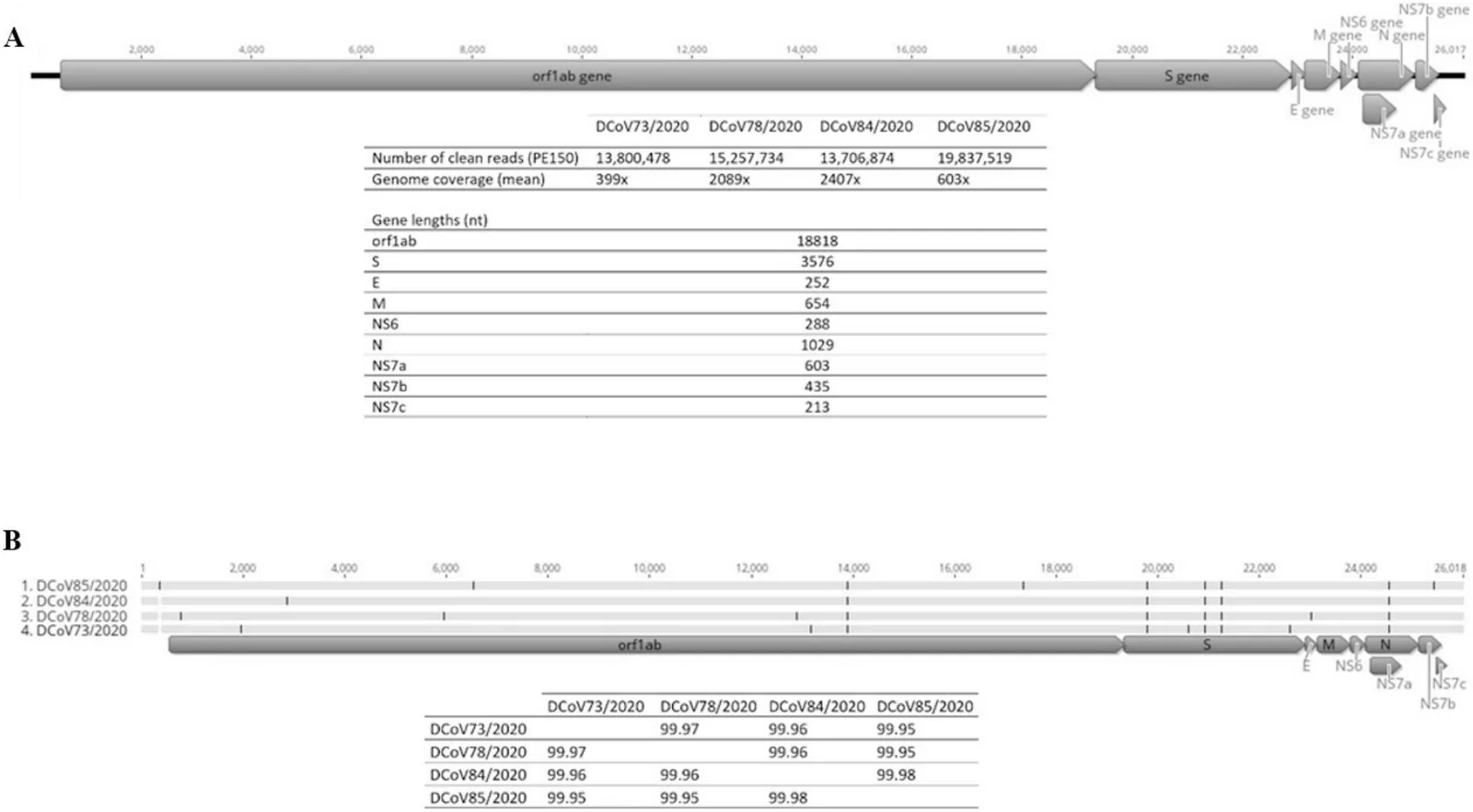
Genome organization and pairwise nucleotide identity of four *Passer montanus* deltacoronavirus (DCoV) strains identified in this study. (A) Schematic genome structure (5′–3′ orientation: ORF1ab–S–E–M–NS6–N–NS7a–NS7b–NS7c). (B) Pairwise genome-wide nucleotide identities (99.95–99.98%).

The genomic architecture (Fig. 1A) follows a 5′–3′ orientation consisting of ORF1ab–S–E–M–NS6–N–NS7a–NS7b–NS7c, consistent with the canonical organization of avian DCoV. Notably, the four genomes showed remarkable conservation, with pairwise nucleotide identities ranging from 99.95% to 99.98% (Fig. 1B), indicating the circulation of a highly homogeneous viral population during the sampling period.

### 3.3 Phylogenetic relationships and gene-specific incongruence

**Fig. 2:**
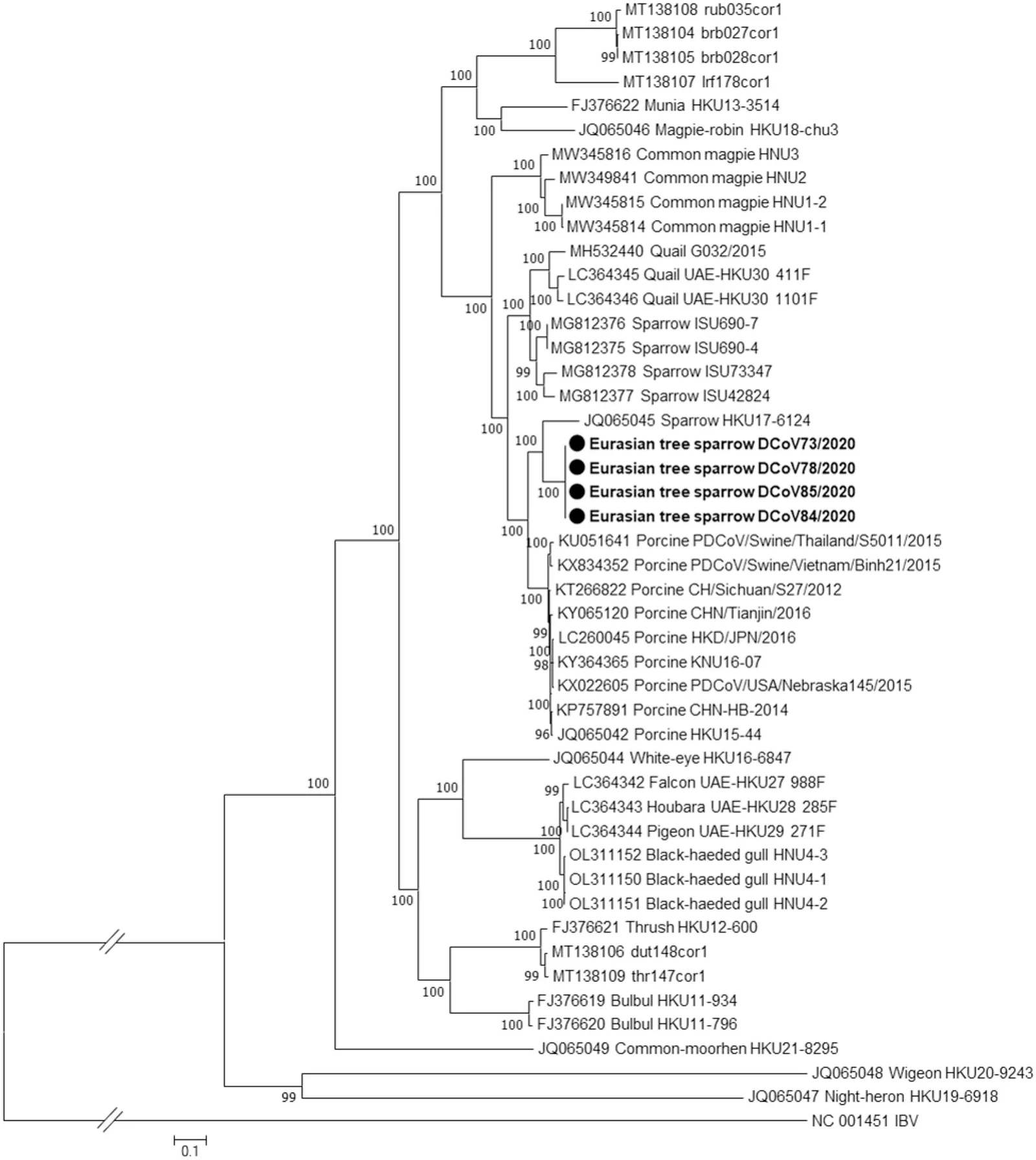
Maximum-likelihood phylogenetic tree of complete DCoV genomes, including four *P. montanus* strains (bold). Bootstrap support (SH-aLRT ≥80%) shown at nodes. Scale bar = substitutions/site.

To determine the evolutionary placement of the novel strains, we conducted a maximum-likelihood phylogenetic analysis of complete genomes (Fig. 2). The four *P. montanus* DCoV strains formed a monophyletic cluster with the avian DCoV strain HKU17–6124 and various porcine DCoVs (PDCoV), sharing genome-wide nucleotide identities of approximately 85.9% and 84.9%, respectively (Table S1).

**Fig. 3:**
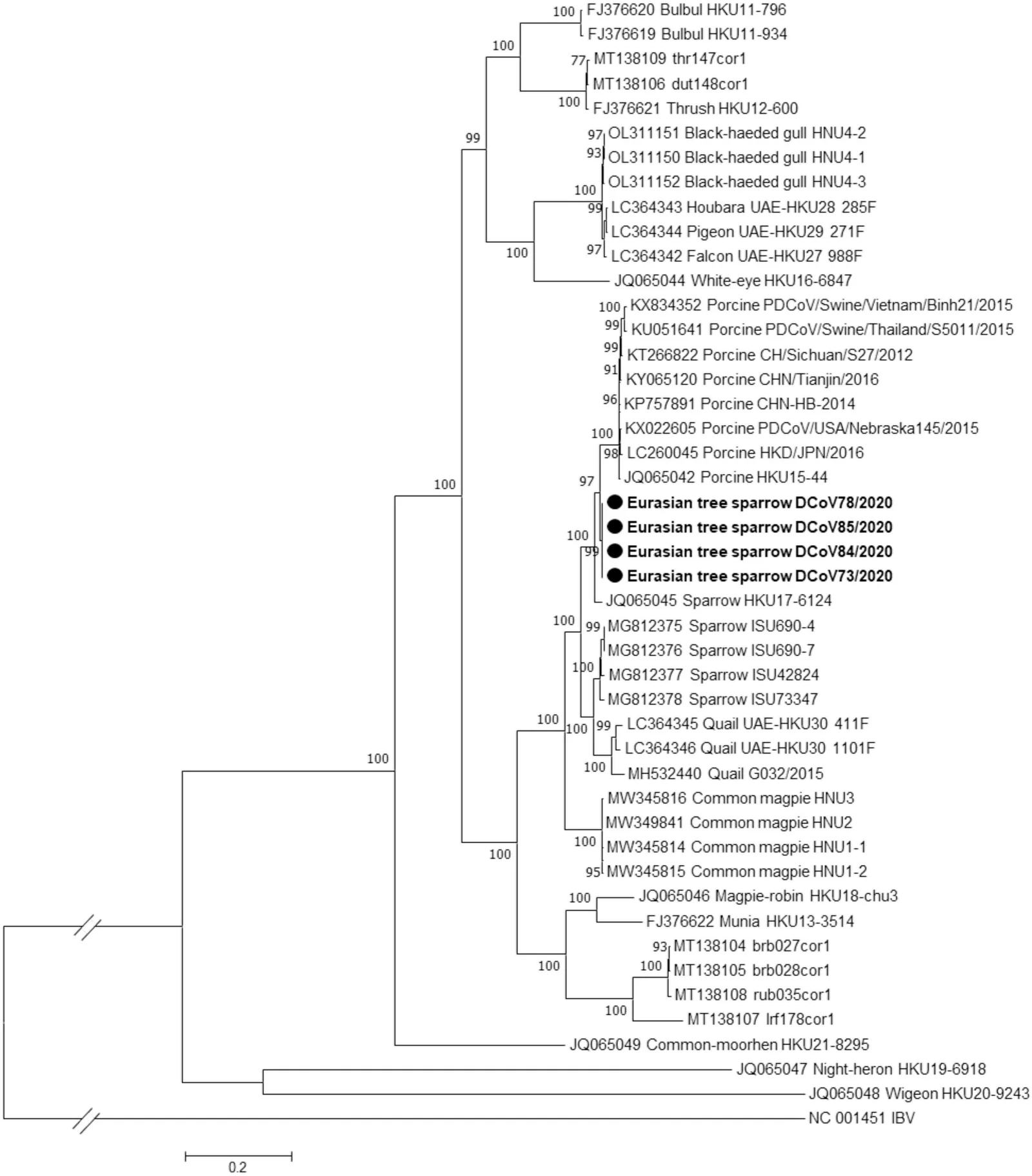
Maximum-likelihood phylogeny based on ORF1ab amino-acid sequences of avian and porcine DCoVs. The *P. montanus* strains clustered with porcine DCoVs (95.6–96.1% aa identity) and sparrow strain HKU17–6124 (97.5% aa identity).

**Fig. 4:**
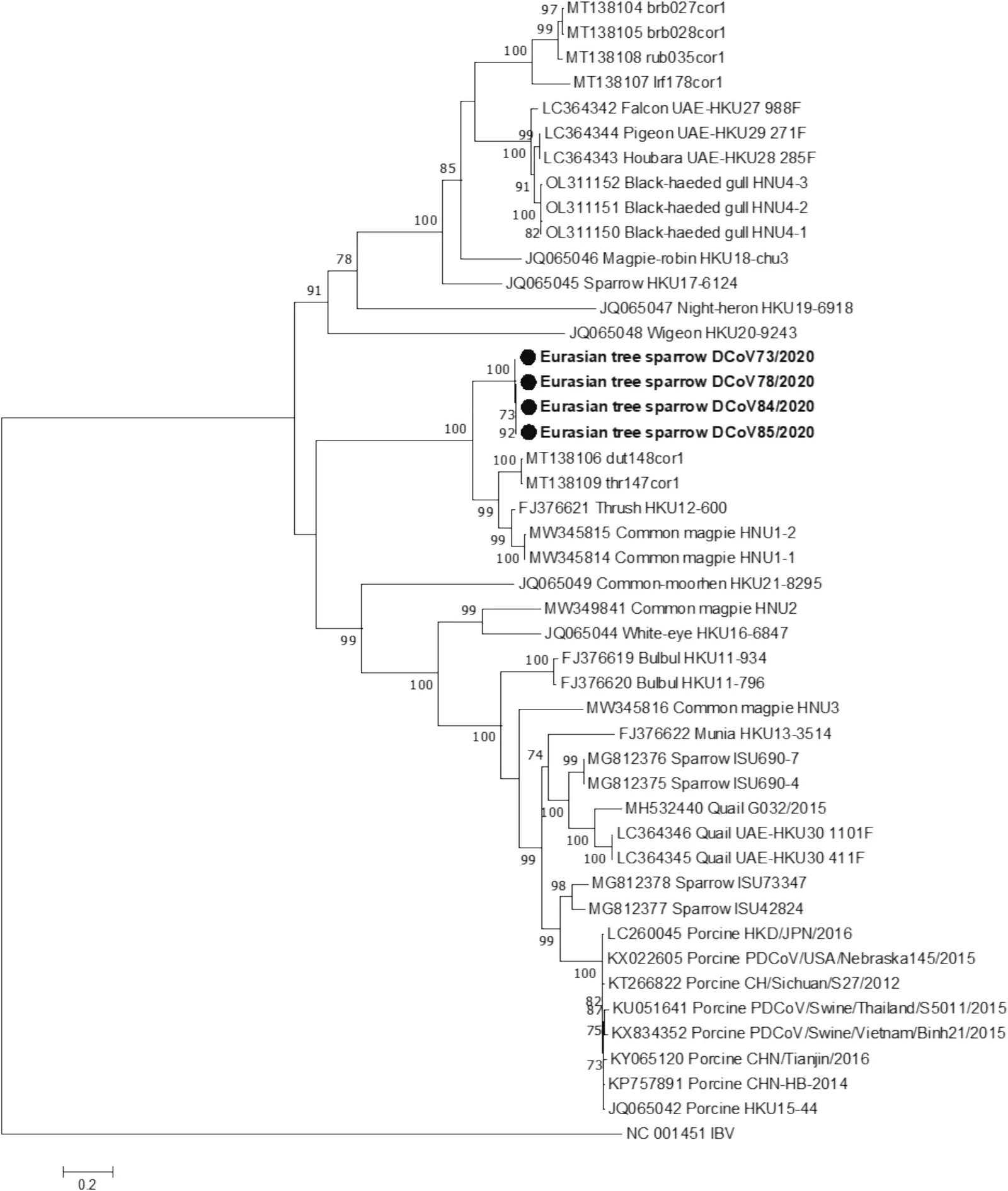
Maximum-likelihood phylogenetic tree of spike (S) protein sequences showing divergence of *P. montanus* DCoVs from porcine DCoVs and grouping with other avian strains (75.7–76.8% aa identity).

However, gene-specific phylogenies revealed marked evolutionary incongruence. In the ORF1ab amino acid tree (Fig. 3), the Slovenian strains clustered robustly within the porcine DCoV clade, exhibiting high identity (95.6%–96.1%) with porcine strains such as HKU15–44 (Table S2). In contrast, the S protein phylogeny (Fig. 4) showed a significant topological shift; here, the *P. montanus* strains diverged completely from the porcine lineage (sharing only ∼47% identity) and instead grouped with a distinct set of avian DCoVs, including strains from thrushes and magpies (75.7%–76.8% identity; Table S3). This pronounced shift in phylogenetic positioning between the replication machinery (ORF1ab) and the entry apparatus (S) provides strong preliminary evidence of a large-scale recombination event.

### 3.4 Identification of a mosaic genome structure

We formally tested for recombination using the RDP5 suite, which identified a single, highly significant recombination event present in all four *P. montanus* genomes. This event was consistently detected by multiple algorithms, with the strongest support provided by the MaxChi method (*p* = 1.17 × 10–43). The analysis mapped a major parental backbone derived from a porcine-like DCoV lineage (represented by strain HK15–44), encompassing the ORF1ab and 3′ terminal regions. This backbone was interrupted by a minor parental insertion spanning the entire Spike (S) gene, most closely related to the avian-origin lineage of strain dut148cor1 (MT138106).

**Fig. 5:**
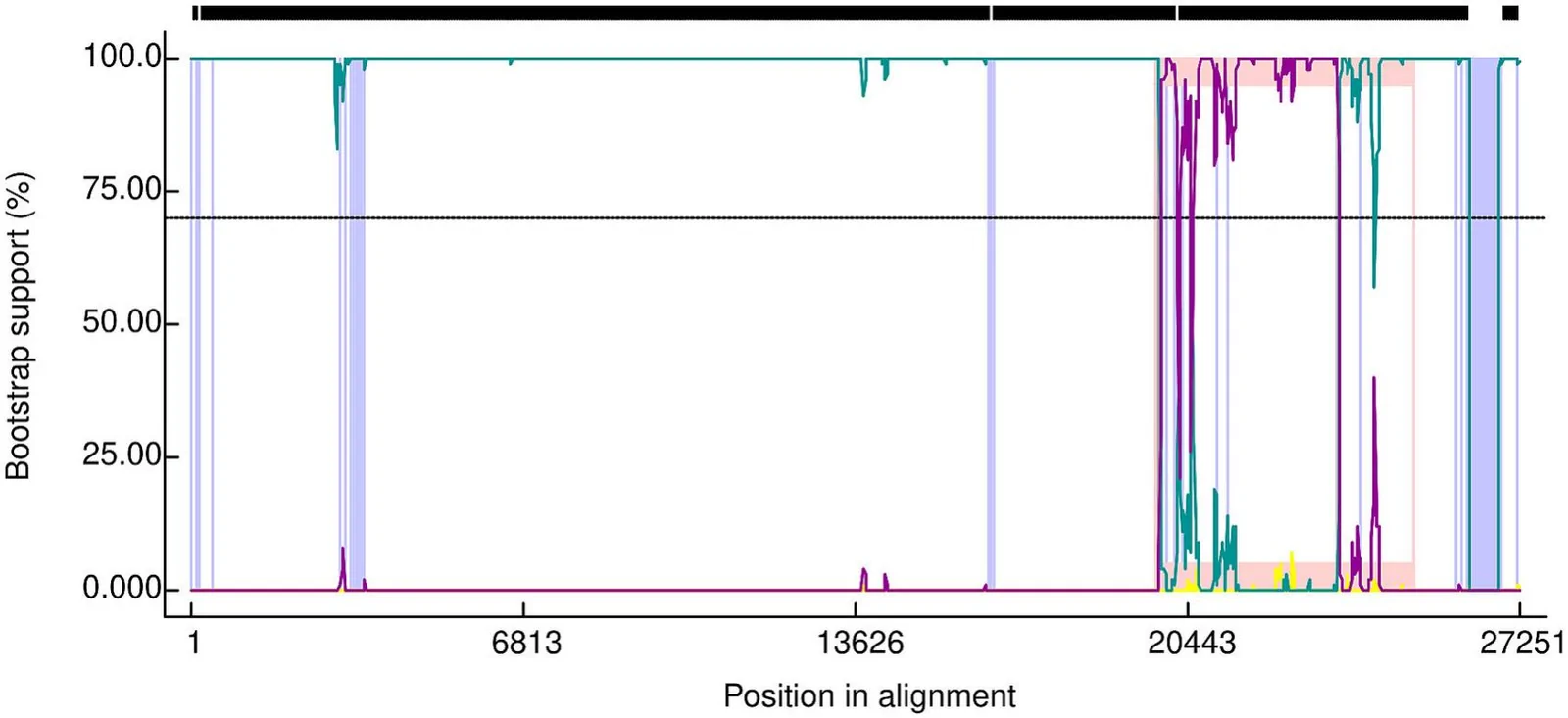
BootScan analysis of the recombination event detected in Eurasian Tree Sparrow DCoV strains. The plot shows bootstrap support across the genome for the four Slovenian isolates (DCoV73/2020, DCoV78/2020, DCoV84/2020, and DCoV85/2020) relative to the inferred major parental strain, porcine DCoV HKU15–44 (JQ065042), and the minor parental strain, avian DCoV dut148cor1 (MT138106). The green/blue curve represents similarity to the porcine parental strain and shows high support across the genomic backbone, with a decline at nucleotide position 19,524. The purple curve represents similarity to the avian parental strain and shows increased support between nucleotide positions 19,524 and approximately 23,000, corresponding to the spike (S) gene region. The yellow curve indicates pairwise sequence similarity between the two parental strains. (For interpretation of the references to colour in this figure legend, the reader is referred to the web version of this article.)

The recombination breakpoints were localised starting at nucleotide position 19,524, creating a mosaic genome that utilises a porcine-origin replication scaffold paired with an avian-origin cell-entry protein. This mosaicism was visually confirmed by BootScan analysis (Fig. 5), which demonstrates a clear shift in phylogenetic affinity at the ORF1ab/S junction.

## 4 Discussion

This study provides the first genomic evidence of DCoVs in Eurasian Tree Sparrows (*Passer montanus*) in Europe, revealing a recombinant DCoV lineage with distinct evolutionary relationships to both avian and porcine DCoVs. Of the 25 *P. montanus* sampled, four were DCoV-positive (16.0%; 95% CI: 4.5%–36.1%), while none of the 218 individuals from 34 other passerine species tested positive. These results indicate species-specific circulation rather than widespread coronavirus prevalence across passerines, emphasising the value of targeted surveillance in synanthropic birds. Although sampling was geographically limited to two sites in Slovenia, *P. montanus* is a widely distributed and synanthropic species with high mobility across Europe, supporting the broader relevance of these findings beyond the immediate sampling area [18], [19].

In Europe, particularly in the Mediterranean, tens of millions of wild passerine birds are illegally trapped each year, often for human consumption [20], [21]. Many are transported along established smuggling routes, including those passing through Slovenia into Italy [35]. These activities facilitate close contact among diverse avian and mammalian hosts and bypass biosecurity controls, increasing the risk of cross-species pathogen transmission and transboundary spread.

Eurasian Tree Sparrows are common in both rural and urban habitats, often foraging in agricultural areas, livestock holdings, and human settlements. The species is recognised as a reservoir or carrier of several pathogens of veterinary and public health significance, including highly pathogenic avian influenza H5N1 [36], Newcastle disease virus [37], *Salmonella enterica* [38], *Chlamydia psittaci* [39], and West Nile virus [40]. Although generally considered short-distance migrants, typically moving within a few hundred kilometres, populations from regions with harsher winter climates have been recorded migrating considerably farther south [41]. During winter in Central Europe, *P. montanus* often coexists with long-distance migratory species such as finches and thrushes, resulting in ecological overlap and potential competition for seasonal food resources [42]. This combination of synanthropy, ecological connectivity, and proven pathogen carriage makes *P. montanus* a relevant species for investigating pathogen circulation at wildlife–livestock interfaces, including the potential role of passerines in DCoV ecology.

Deltacoronaviruses were first identified in wild birds by [10], and subsequent surveillance has revealed increasing diversity among avian and mammalian DCoVs worldwide [10], [43], [44], [45]. Porcine DCoV (PDCoV) emerged in pigs in North America and Asia in 2014 and has since been reported in multiple countries, with molecular and evolutionary evidence suggesting an avian origin [7], [10], [16]. Our phylogenetic analysis revealed distinct patterns of relatedness among genomic regions. In the ORF1ab tree, the four *P. montanus* DCoV strains formed a strongly supported cluster with porcine DCoVs and the avian sparrow strain HKU17–6124, sharing high amino acid identities (95.6%–96.1% with porcine DCoVs; 97.5% with HKU17–6124). This close similarity in the replicase region suggests either recent divergence from a common ancestor or historical interspecies viral exchange, consistent with previous hypotheses of avian-to-mammalian spillover in DCoV evolution [7], [10], [16].

In contrast, the spike (S) gene phylogeny showed significantly lower amino acid identities to PDCoVs (approximately 47%) and instead grouped the *P. montanus* strains with a different set of avian viruses, including dut148cor, thr147cor1, Thrush_HKU12–600, and common magpie (HNU1) strains (75.7%–76.8% identity). As the spike protein is the primary determinant of host range and cell entry [46], this divergence reflects adaptation to an avian host or circulation within a previously unrecognised lineage of wild passerines.

This incongruence between the ORF1ab and S gene phylogenies is consistent with patterns observed in coronaviruses, where both recombination and lineage-specific divergence drive evolution [17], [47]. Although lineage-specific divergence is always present, our RDP5 and BootScan analyses provide definitive evidence of modular recombination. In infectious bronchitis virus and other avian CoVs, the spike gene is known to undergo such modular recombination [13], enabling rapid adaptation to new hosts while preserving the conserved replication machinery in ORF1ab. Our findings show that *P. montanus* DCoVs have employed this evolutionary strategy to generate a mosaic genome.

The observed divergence of the spike gene from both porcine and other avian DCoVs suggests that the detected viruses represent a distinct evolutionary lineage with a unique combination of genomic features. This specific genomic pattern – a highly conserved ORF1ab related to porcine DCoVs combined with a divergent avian-associated S gene – highlights the evolutionary connectivity among DCoVs associated with different host groups and the potential role of recombination in facilitating changes in host association. The ecological overlap of wild birds, domestic poultry, pigs, and humans – particularly in settings with informal animal trade, mixed-species holdings, or low biosecurity – creates opportunities for viral exchange and potential recombination events [3], [10], [48], [49]. Such scenarios support the inclusion of passerines, especially synanthropic species such as *P. montanus*, in broader coronavirus surveillance at wildlife–livestock interfaces.

Although this study involved a limited number of individuals and relied on opportunistic sampling, it provides the first genomic evidence of DCoV circulation in *P. montanus* in Europe, establishing an important baseline for future surveillance. Broader geographic and temporal sampling – ideally combined with in vivo or in vitro studies of host–virus interactions – will be essential to assess replication competence, receptor usage, and spillover potential. Integrating ecological, virological, and genomic approaches across multiple taxa would substantially improve our understanding of how avian DCoVs evolve and persist at the wildlife–livestock interface. Furthermore, while pooled screening was used to optimise assay efficiency, followed by individual testing of positive pools, the possibility of false negatives in samples with low viral loads cannot be excluded.

At the European level, coordinated One Health surveillance initiatives increasingly recognise the importance of wildlife surveillance and of integrating wildlife monitoring with pathogen surveillance [50]. However, current European wildlife surveillance frameworks focus predominantly on established priority pathogens, while coronavirus surveillance in passerines remains limited.

## 5 Conclusion

In conclusion, this study provides the first genomic characterisation of DCoVs in Eurasian Tree Sparrows (*Passer montanus*) in Europe. Our findings demonstrate distinct evolutionary relationships with both porcine and avian DCoVs and provide strong evidence of a large-scale modular recombination event (*p* = 1.17 × 10–43), with the ORF1ab region closely related to porcine DCoVs and the spike gene clustering with divergent avian DCoVs. The synanthropic ecology and mobility of *P. montanus*, together with the detection of these recombinant viruses, identify this species as a relevant target for further investigation of the ecological connectivity of DCoVs across wildlife and domestic-animal interfaces. These results support the inclusion of passerines in broader coronavirus surveillance frameworks and demonstrate the value of genomic surveillance for identifying recombinant viruses and improving understanding of DCoV evolution across wildlife–livestock interfaces.

## Ethic statement

The handling of wild birds was approved by the Slovenian Environment Agency (permit no. 35601–8/2015–7). No birds were captured or handled solely for the purposes of this study. Fresh fecal samples were collected opportunistically during routine bird ringing activities as part of the regular monitoring of migratory birds in Slovenia during the European autumn migration periods of 2020 and 2021. All birds were captured using mist nets by licensed ringers, handled in compliance with national regulations, and released immediately after ringing. Fecal material was collected only if defecation occurred during handling, without causing additional distress.

## Animal subject

This study was conducted in accordance with the ARRIVE (Animal Research: Reporting of In Vivo Experiments) guidelines.

This study was approved by the Slovenian Environment Agency.

(Approval No. permit no. 35601–8/2015–7)

## CRediT authorship contribution statement

**Urška Kuhar:** Writing – review & editing, Writing – original draft, Validation, Supervision, Resources, Methodology, Investigation, Funding acquisition, Formal analysis, Data curation, Conceptualization. **Neža Mrzdovnik:** Writing – review & editing, Writing – original draft, Investigation. **Danijela Černe:** Writing – review & editing, Validation, Software, Investigation, Formal analysis. **Petra Šenica Kavčič:** Writing – review & editing, Validation, Software, Investigation, Formal analysis. **Tomi Trilar:** Writing – review & editing, Supervision, Resources, Project administration, Funding acquisition, Data curation. **Milka Vrecl Fazarinc:** Writing – review & editing, Visualization, Supervision, Methodology, Funding acquisition, Conceptualization. **Modest Vengust:** Writing – review & editing, Writing – original draft, Visualization, Supervision, Resources, Project administration, Methodology, Investigation, Funding acquisition, Formal analysis, Data curation, Conceptualization.

## Funding sources

The authors gratefully acknowledge financial support from the 10.13039/501100004329Slovenian Research Agency (core funding nos. P4–0053, P4–0455, and P4–0092) and from the Slovenian Museum of Natural History (Stable funding of scientific research activities, grant no. 0614).

## Declaration of competing interest

The authors declare the following financial interests/personal relationships which may be considered as potential competing interests: Modest Vengust reports financial support was provided by Slovenian Research and Innovation Agency. If there are other authors, they declare that they have no known competing financial interests or personal relationships that could have appeared to influence the work reported in this paper.

## Data Availability

Data will be made available on request.
