# Supplementary material for: Coronavirus surveillance in passerines reveals novel deltacoronaviruses in Eurasian tree sparrows with implications for One Health and livestock biosecurity
Source: One Health. 2026 Aug 31;23:101560. doi: 10.1016/j.onehlt.2026.101560 (PMC13563232; doi:10.1016/j.onehlt.2026.101560)
Supplement: Supplementary file 1 [file mmc1.docx]

**Supplementary Tables S1–S3.**

Pairwise sequence identity matrices for deltacoronavirus (DCoV) genomes and major coding regions.

**Table S1**: Pairwise percent nucleotide (% nt) identities among complete DCoV genomes, including four *Passer montanus* strains (**bold**) and selected avian and porcine references. Values were derived from whole-genome alignments used to generate Figure 2.

**Table S2**: Pairwise percent amino-acid (% aa) identities in the ORF1ab polyprotein between four *P. montanus* DCoVs and representative avian and porcine strains. Values correspond to alignments used for Figure 3 (ORF1ab phylogeny).

**Table S3:** Pairwise percent amino-acid (% aa) identities of the spike (S) protein among four *P. montanus* DCoVs and selected avian and porcine references. Values correspond to alignments used in Figure 4 (spike phylogeny).

Table S1

|  | % nt identity | | | |
| --- | --- | --- | --- | --- |
|  | DCoV85/2020 | DCoV84/2020 | DCoV78/2020 | CoV73/2020 |
| ***Eurasian tree sparrow* DCoV85/2020** |  | **99.98** | **99.95** | **99.95** |
| ***Eurasian tree sparrow* DCoV84/2020** | **99.98** |  | **99.96** | **99.96** |
| ***Eurasian tree sparrow* DCoV78/2020** | **99.95** | **99.96** |  | **99.97** |
| ***Eurasian tree sparrow* DCoV73/2020** | **99.95** | **99.96** | **99.97** |  |
| JQ065045_Sparrow_HKU17-6124 | 85.87 | 85.87 | 85.87 | 85.89 |
| JQ065042_Porcine_HKU15-44 | 84.90 | 84.91 | 84.90 | 84.92 |
| KP757891_Porcine_CHN-HB-2014 | 84.94 | 84.95 | 84.94 | 84.95 |
| KY364365_Porcine_KNU16-07 | 84.84 | 84.85 | 84.84 | 84.86 |
| KX022605_Porcine_PDCoV/USA/Nebraska145/2015 | 84.86 | 84.86 | 84.86 | 84.87 |
| LC260045_Porcine_HKD/JPN/2016 | 84.66 | 84.66 | 84.66 | 84.67 |
| KT266822_Porcine_CH/Sichuan/S27/2012 | 84.88 | 84.88 | 84.88 | 84.89 |
| KY065120_Porcine_CHN/Tianjin/2016 | 84.69 | 84.70 | 84.69 | 84.70 |
| KX834352_Porcine_PDCoV/Swine/Vietnam/Binh21/2015 | 84.65 | 84.66 | 84.65 | 84.67 |
| KU051641_Porcine_PDCoV/Swine/Thailand/S5011/2015 | 84.49 | 84.49 | 84.49 | 84.50 |
| MG812376_Sparrow_ISU690-7 | 81.38 | 81.38 | 81.37 | 81.38 |
| MG812375_Sparrow_ISU690-4 | 81.39 | 81.39 | 81.38 | 81.39 |
| MG812377_Sparrow_ISU42824 | 81.39 | 81.39 | 81.39 | 81.40 |
| MG812378_Sparrow_ISU73347 | 81.45 | 81.46 | 81.44 | 81.45 |
| MH532440_Quail_G032/2015 | 79.18 | 79.18 | 79.17 | 79.18 |
| LC364345_Quail_UAE-HKU30_411F | 79.37 | 79.38 | 79.36 | 79.37 |
| LC364346_Quail_UAE-HKU30_1101F | 79.41 | 79.41 | 79.39 | 79.42 |
| MW345815_Common_magpie_HNU1-2 | 79.01 | 79.02 | 79.00 | 78.99 |
| MW345814_Common_magpie_HNU1-1 | 79.02 | 79.03 | 79.01 | 79.00 |
| MW349841_Common_magpie_HNU2 | 77.41 | 77.41 | 77.40 | 77.40 |
| MW345816_Common_magpie_HNU3 | 77.45 | 77.45 | 77.43 | 77.44 |
| JQ065046_Magpie-robin_HKU18-chu3 | 67.61 | 67.61 | 67.59 | 67.59 |
| FJ376622_Munia_HKU13-3514 | 67.86 | 67.86 | 67.85 | 67.86 |
| FJ376621_Thrush_HKU12-600 | 67.31 | 67.31 | 67.30 | 67.29 |
| MT138106_dut148cor1 | 67.11 | 67.11 | 67.11 | 67.11 |
| MT138109_thr147cor1 | 67.03 | 67.03 | 67.02 | 67.03 |
| MT138105_brb028cor1 | 65.79 | 65.78 | 65.78 | 65.78 |
| MT138104_brb027cor1 | 65.78 | 65.78 | 65.77 | 65.77 |
| MT138108_rub035cor1 | 65.61 | 65.61 | 65.61 | 65.61 |
| MT138107_lrf178cor1 | 65.28 | 65.29 | 65.28 | 65.29 |
| FJ376619_Bulbul_HKU11-934 | 65.57 | 65.57 | 65.56 | 65.57 |
| FJ376620_Bulbul_HKU11-796 | 65.60 | 65.59 | 65.58 | 65.60 |
| LC364342_Falcon_UAE-HKU27_988F | 65.31 | 65.32 | 65.31 | 65.32 |
| LC364344_Pigeon_UAE-HKU29_271F | 65.19 | 65.19 | 65.20 | 65.21 |
| LC364343_Houbara_UAE-HKU28_285F | 65.18 | 65.17 | 65.18 | 65.20 |
| OL311152_Black-headed_gull_HNU4-3 | 65.32 | 65.33 | 65.32 | 65.33 |
| OL311151_Black-headed_gull_HNU4-2 | 65.32 | 65.32 | 65.32 | 65.33 |
| OL311150_Black-headed_gull_HNU4-1 | 65.32 | 65.33 | 65.32 | 65.33 |
| JQ065044_White-eye_HKU16-6847 | 65.08 | 65.07 | 65.06 | 65.08 |
| JQ065049_Common-moorhen_HKU21-8295 | 59.46 | 59.45 | 59.46 | 59.46 |
| JQ065047_Night-heron_HKU19-6918 | 51.77 | 51.76 | 51.79 | 51.78 |
| JQ065048_Wigeon_HKU20-9243 | 50.65 | 50.64 | 50.64 | 50.65 |
| NC_001451_IBV | 40.17 | 40.16 | 40.15 | 40.15 |

Table S2

|  | % amino-acid identity (ORF1ab polyprotein) | | | |
| --- | --- | --- | --- | --- |
|  | DCoV73/2020 | DCoV78/2020 | DCoV84/2020 | DCoV85/2020 |
| Eurasian_tree_sparrow_DCoV73/2020 |  | 100.00 | 100.00 | 99.98 |
| Eurasian_tree_sparrow_DCoV78/2020 | 100.00 |  | 100.00 | 99.98 |
| Eurasian_tree_sparrow_DCoV84/2020 | 100.00 | 100.00 |  | 99.98 |
| Eurasian_tree_sparrow_DCoV85/2020 | 99.98 | 99.98 | 99.98 |  |
| JQ065045_Sparrow_HKU17-6124 | 97.52 | 97.52 | 97.52 | 97.50 |
| JQ065042_Porcine_HKU15-44 | 96.10 | 96.10 | 96.10 | 96.08 |
| KP757891_Porcine_CHN-HB-2014 | 96.12 | 96.12 | 96.12 | 96.10 |
| KX022605_Porcine_PDCoV/USA/Nebraska145/2015 | 95.94 | 95.94 | 95.94 | 95.92 |
| LC260045_Porcine_HKD/JPN/2016 | 95.73 | 95.73 | 95.73 | 95.72 |
| KY065120_Porcine_CHN/Tianjin/2016 | 95.88 | 95.88 | 95.88 | 95.86 |
| KT266822_Porcine_CH/Sichuan/S27/2012 | 95.94 | 95.94 | 95.94 | 95.92 |
| KU051641_Porcine_PDCoV/Swine/Thailand/S5011/2015 | 95.65 | 95.65 | 95.65 | 95.64 |
| KX834352_Porcine_PDCoV/Swine/Vietnam/Binh21/2015 | 95.70 | 95.70 | 95.70 | 95.68 |
| MG812375_Sparrow_ISU690-4 | 93.34 | 93.34 | 93.34 | 93.33 |
| MG812376_Sparrow_ISU690-7 | 93.34 | 93.34 | 93.34 | 93.33 |
| MG812377_Sparrow_ISU42824 | 93.21 | 93.21 | 93.21 | 93.20 |
| MG812378_Sparrow_ISU73347 | 93.17 | 93.17 | 93.17 | 93.15 |
| LC364345_Quail_UAE-HKU30_411F | 91.14 | 91.14 | 91.14 | 91.12 |
| LC364346_Quail_UAE-HKU30_1101F | 91.17 | 91.17 | 91.17 | 91.15 |
| MH532440_Quail_G032/2015 | 90.72 | 90.72 | 90.72 | 90.70 |
| MW345814_Common_magpie_HNU1-1 | 89.15 | 89.15 | 89.15 | 89.14 |
| MW345815_Common_magpie_HNU1-2 | 89.12 | 89.12 | 89.12 | 89.11 |
| MW349841_Common_magpie_HNU2 | 89.20 | 89.20 | 89.20 | 89.19 |
| MW345816_Common_magpie_HNU3 | 89.03 | 89.03 | 89.03 | 89.01 |
| JQ065046_Magpie-robin_HKU18-chu3 | 79.88 | 79.88 | 79.88 | 79.88 |
| FJ376622_Munia_HKU13-3514 | 78.74 | 78.74 | 78.74 | 78.74 |
| MT138104_brb027cor1 | 77.74 | 77.74 | 77.74 | 77.74 |
| MT138105_brb028cor1 | 77.76 | 77.76 | 77.76 | 77.76 |
| MT138108_rub035cor1 | 77.57 | 77.57 | 77.57 | 77.57 |
| MT138107_lrf178cor1 | 76.85 | 76.85 | 76.85 | 76.85 |
| FJ376619_Bulbul_HKU11-934 | 75.76 | 75.76 | 75.76 | 75.76 |
| FJ376620_Bulbul_HKU11-796 | 75.84 | 75.84 | 75.84 | 75.84 |
| FJ376621_Thrush_HKU12-600 | 75.46 | 75.46 | 75.46 | 75.46 |
| MT138106_dut148cor1 | 75.51 | 75.51 | 75.51 | 75.51 |
| MT138109_thr147cor1 | 75.40 | 75.40 | 75.40 | 75.40 |
| OL311150_Black-headed_gull_HNU4-1 | 75.16 | 75.16 | 75.16 | 75.16 |
| OL311151_Black-headed_gull_HNU4-2 | 75.16 | 75.16 | 75.16 | 75.16 |
| OL311152_Black-headed_gull_HNU4-3 | 75.13 | 75.13 | 75.13 | 75.13 |
| LC364342_Falcon_UAE-HKU27_988F | 74.97 | 74.97 | 74.97 | 74.97 |
| LC364343_Houbara_UAE-HKU28_285F | 74.91 | 74.91 | 74.91 | 74.91 |
| LC364344_Pigeon_UAE-HKU29_271F | 74.92 | 74.92 | 74.92 | 74.92 |
| JQ065044_White-eye_HKU16-6847 | 73.84 | 73.84 | 73.84 | 73.84 |
| JQ065049_Common-moorhen_HKU21-8295 | 69.12 | 69.12 | 69.12 | 69.12 |
| JQ065047_Night-heron_HKU19-6918 | 51.52 | 51.52 | 51.52 | 51.52 |
| JQ065048_Wigeon_HKU20-9243 | 49.95 | 49.95 | 49.95 | 49.95 |
| NC_001451_IBV | 33.83 | 33.83 | 33.83 | 33.83 |

Table S3

|  | % amino-acid identity (spike protein) | | | |
| --- | --- | --- | --- | --- |
|  | DCoV73/2020 | DCoV78/2020 | DCoV84/2020 | DCoV85/2020 |
| Eurasian_tree_sparrow_DCoV73/2020 |  | 99.92 | 99.75 | 99.75 |
| Eurasian_tree_sparrow_DCoV78/2020 | 99.92 |  | 99.83 | 99.83 |
| Eurasian_tree_sparrow_DCoV84/2020 | 99.75 | 99.83 |  | 100.00 |
| Eurasian_tree_sparrow_DCoV85/2020 | 99.75 | 99.83 | 100.00 |  |
| FJ376621_Thrush_HKU12-600 | 76.81 | 76.81 | 76.73 | 76.73 |
| MW345814_Common_magpie_HNU1-1 | 75.50 | 75.50 | 75.42 | 75.42 |
| MW345815_Common_magpie_HNU1-2 | 75.42 | 75.42 | 75.33 | 75.33 |
| MT138106_dut148cor1 | 75.63 | 75.63 | 75.54 | 75.54 |
| MT138109_thr147cor1 | 75.71 | 75.71 | 75.63 | 75.63 |
| MG812377_Sparrow_ISU42824 | 47.14 | 47.14 | 47.05 | 47.05 |
| MG812378_Sparrow_ISU73347 | 48.01 | 48.01 | 47.93 | 47.93 |
| JQ065042_Porcine_HKU15-44 | 47.10 | 47.10 | 47.02 | 47.02 |
| KX022605_Porcine_PDCoV/USA/Nebraska145/2015 | 47.02 | 47.02 | 46.94 | 46.94 |
| KP757891_Porcine_CHN-HB-2014 | 47.02 | 47.02 | 46.94 | 46.94 |
| LC260045_Porcine_HKD/JPN/2016 | 46.77 | 46.77 | 46.69 | 46.69 |
| KT266822_Porcine_CH/Sichuan/S27/2012 | 46.94 | 46.94 | 46.85 | 46.85 |
| KY065120_Porcine_CHN/Tianjin/2016 | 46.78 | 46.78 | 46.69 | 46.69 |
| KX834352_Porcine_PDCoV/Swine/Vietnam/Binh21/2015 | 46.52 | 46.52 | 46.44 | 46.44 |
| KU051641_Porcine_PDCoV/Swine/Thailand/S5011/2015 | 47.10 | 47.10 | 47.02 | 47.02 |
| MG812375_Sparrow_ISU690-4 | 46.51 | 46.51 | 46.43 | 46.43 |
| MG812376_Sparrow_ISU690-7 | 46.51 | 46.51 | 46.43 | 46.43 |
| LC364345_Quail_UAE-HKU30_411F | 46.24 | 46.24 | 46.15 | 46.15 |
| LC364346_Quail_UAE-HKU30_1101F | 46.24 | 46.24 | 46.15 | 46.15 |
| MH532440_Quail_G032/2015 | 45.79 | 45.79 | 45.71 | 45.71 |
| MW345816_Common_magpie_HNU3 | 46.29 | 46.29 | 46.12 | 46.12 |
| FJ376619_Bulbul_HKU11-934 | 45.79 | 45.79 | 45.71 | 45.71 |
| FJ376620_Bulbul_HKU11-796 | 45.58 | 45.58 | 45.50 | 45.50 |
| FJ376622_Munia_HKU13-3514 | 45.93 | 45.93 | 45.85 | 45.85 |
| JQ065044_White-eye_HKU16-6847 | 44.57 | 44.57 | 44.49 | 44.49 |
| MW349841_Common_magpie_HNU2 | 45.70 | 45.70 | 45.62 | 45.62 |
| JQ065049_Common-moorhen_HKU21-8295 | 44.61 | 44.61 | 44.53 | 44.53 |
| JQ065045_Sparrow_HKU17-6124 | 45.61 | 45.61 | 45.53 | 45.53 |
| JQ065046_Magpie-robin_HKU18-chu3 | 42.50 | 42.50 | 42.42 | 42.42 |
| LC364342_Falcon_UAE-HKU27_988F | 42.95 | 42.95 | 42.87 | 42.87 |
| LC364343_Houbara_UAE-HKU28_285F | 43.35 | 43.35 | 43.27 | 43.27 |
| LC364344_Pigeon_UAE-HKU29_271F | 43.35 | 43.35 | 43.27 | 43.27 |
| OL311150_Black-headed_gull_HNU4-1 | 43.00 | 43.00 | 42.91 | 42.91 |
| OL311151_Black-headed_gull_HNU4-2 | 43.00 | 43.00 | 42.91 | 42.91 |
| OL311152_Black-headed_gull_HNU4-3 | 43.08 | 43.08 | 43.00 | 43.00 |
| MT138104_brb027cor1 | 43.34 | 43.34 | 43.26 | 43.26 |
| MT138105_brb028cor1 | 43.34 | 43.34 | 43.26 | 43.26 |
| MT138108_rub035cor1 | 43.42 | 43.42 | 43.34 | 43.34 |
| MT138107_lrf178cor1 | 43.83 | 43.83 | 43.74 | 43.74 |
| JQ065048_Wigeon_HKU20-9243 | 40.47 | 40.47 | 40.39 | 40.39 |
| JQ065047_Night-heron_HKU19-6918 | 40.74 | 40.74 | 40.67 | 40.67 |
| NC_001451_IBV | 24.43 | 24.43 | 24.35 | 24.35 |
